# Gigantic Stomach: A Rare Manifestation of Duchenne Muscular Dystrophy

**DOI:** 10.7759/cureus.4609

**Published:** 2019-05-07

**Authors:** Amaninder Dhaliwal, Sarvani Madiraju, Banreet S Dhindsa, Getaw W Hassen, Fedja A Rochling

**Affiliations:** 1 Gastroenterology, University of Nebraska Medical Center, Omaha, USA; 2 Miscellaneous, St. George's University, St. George, GRD; 3 Internal Medicine, University of Nevada School of Medicine, Las Vegas, USA; 4 Emergency Medicine, New York Medical College and Metropolitan Hospital Center, New York, USA

**Keywords:** duchenne muscular dystrophy, gastroparesis, gastric dilatation, constipation

## Abstract

Duchenne muscular dystrophy (DMD) is characterized by degeneration and atrophy of skeletal, cardiac, and smooth muscles after a latent period of apparently normal development and function. The gastrointestinal manifestations start in the second decade of life and are mainly due to atrophy of smooth muscle layers.^ ^Refractory gastroparesis and chronic constipation can lead to severe gastric and small bowel dilatation, which can be life threatening. Here, we present a case of a 21-year-old male with a gigantic stomach secondary to DMD resolved with conservative management and no surgical intervention.

## Introduction

Duchenne muscular dystrophy (DMD) is characterized by degeneration and atrophy of skeletal, cardiac, and smooth muscles after a latent period of apparently normal development and function. The gastrointestinal manifestations start in the second decade of life and are mainly due to atrophy of smooth muscle layers [1]. Gastrointestinal manifestations in DMD are not well studied and can vary in severity. Refractory gastroparesis can lead to severe gastric and small bowel dilatation. Chronic constipation can further compound the severity of gastric or small bowel dilatation, which can be life threatening. The treatment of these manifestations is mainly conservative with medical management and rarely requires surgery. Few case reports of severe gastric and small bowel dilatation in DMD patients have been reported in the literature. Here, we present a case of a 21-year-old male with gigantic stomach secondary to DMD resolved with conservative management and no surgical intervention.

## Case presentation

A 21-year-old male with a past medical history of DMD, scoliosis with multiple back surgeries, failure to thrive and atrial fibrillation presented with abdominal pain and vomiting for two days. He was chronically constipated since the age of 15 years and had one to two bowel movements per month with no use of laxatives. On admission, the patient was afebrile, tachycardic with a heart rate of 148 beats per minute, hypotensive with a blood pressure of 89/55 mmHg, and tachypneic with a respiratory rate of 36/min. He was cachectic with severe muscle wasting and had dry mucous membranes. His body mass index (BMI) was 14.8 kg/m2. Physical exam showed a soft, non-tender, distended abdomen, with no guarding and rigidity. The patient presented with hypoactive bowel sounds and chronic muscle contractions in all the four extremities. Laboratory values showed leukocytosis 20300/µL (normal 4000-10,000/µL) with left shift (bands 22), hemoglobin 16.4 g/dL (normal 14-17 g/dL) and hematocrit 51.1% (normal 41%-51%), blood urea nitrogen (BUN) 31 mg/dL (normal 8-20 mg/dL), creatinine 0.40 mg/dL (normal 0.7-1.3 mg/dL), mild elevation of aspartate aminotransferase (AST) 54 IU/L (normal 40-35 U/L), prothrombin time (PT) 14s (normal 11-13 s), international normalized ratio (INR) 1.3 (normal <1.1), partial thromboplastin time (PTT) 32.3 (normal 25-35 s) and normal albumin 4.7 g/dL (normal 3.5-5.5 g/dL). He was severely intravascular volume-depleted as per physical exam and laboratory values. He received aggressive fluid resuscitation with Ringer’s lactate.

**Figure 1:**
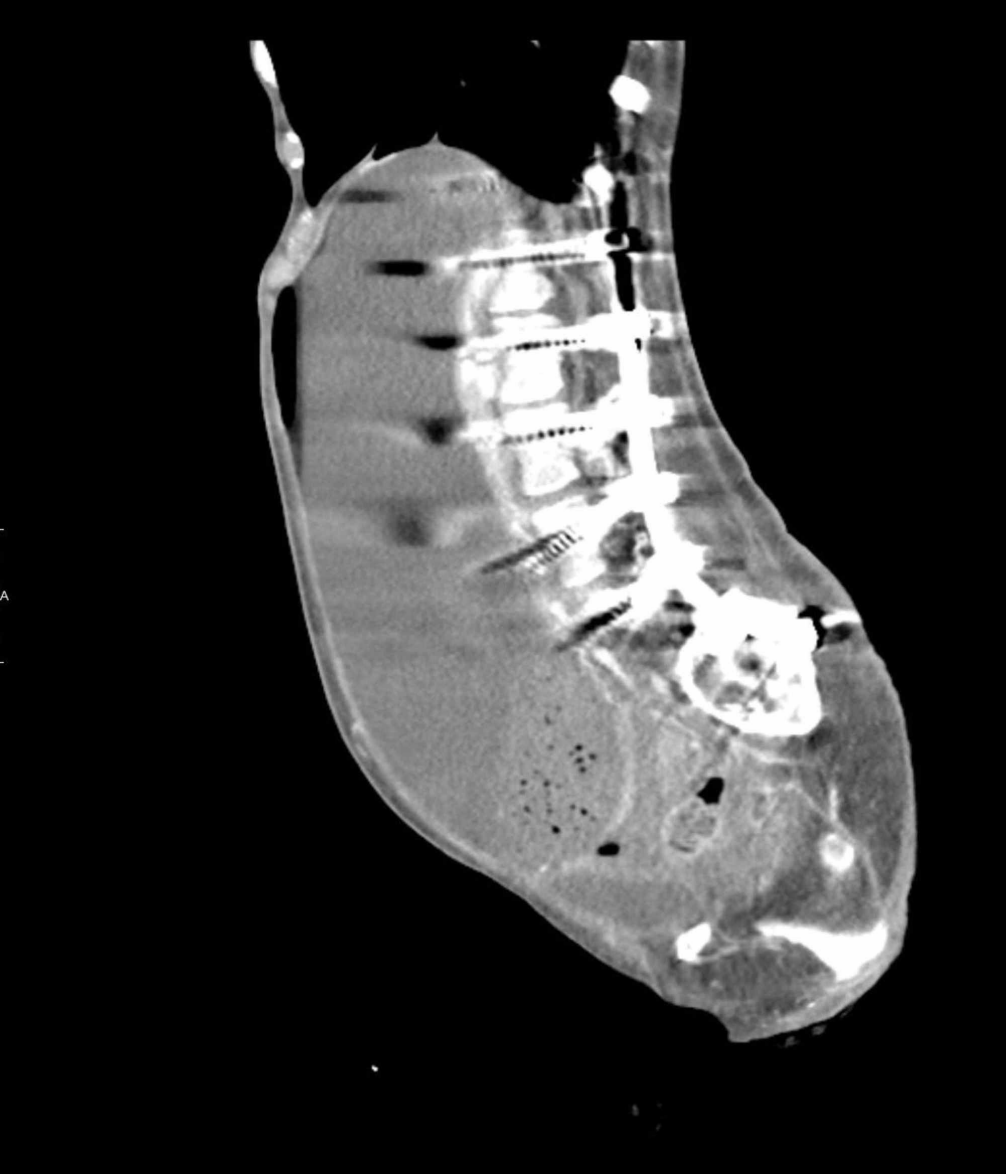
Computed tomography (CT) scan of the abdomen (sagittal section) with contrast showing massively distended stomach extending into the pelvis

**Figure 2:**
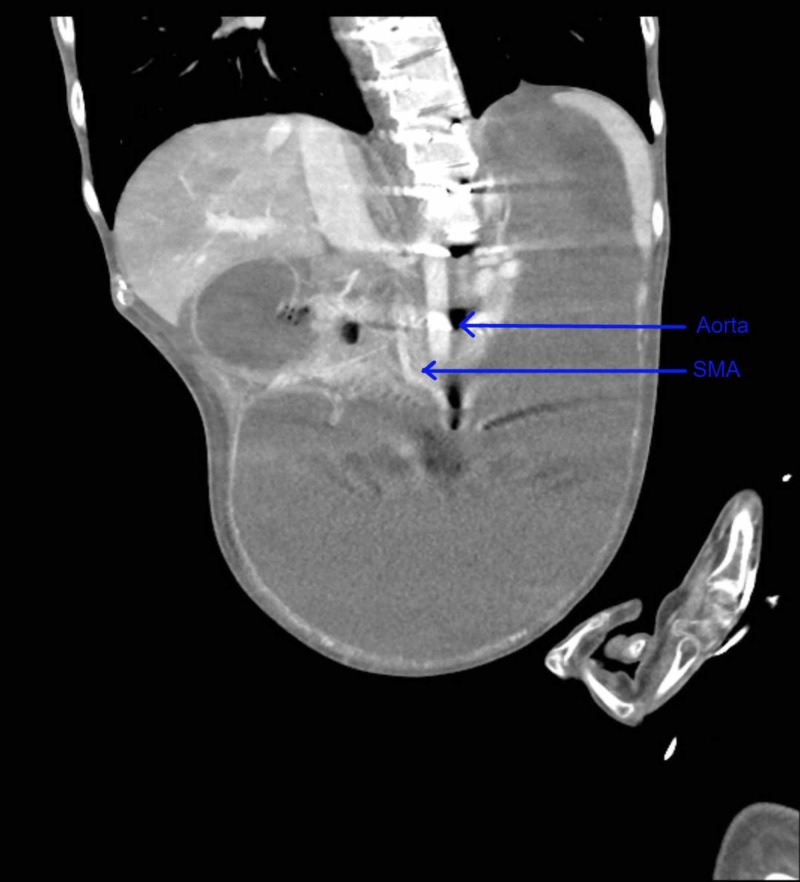
Computed tomography (CT) scan of the abdomen (coronal section) with contrast showing massively distended stomach extending into the pelvis SMA: superior mesenteric artery.

**Figure 3:**
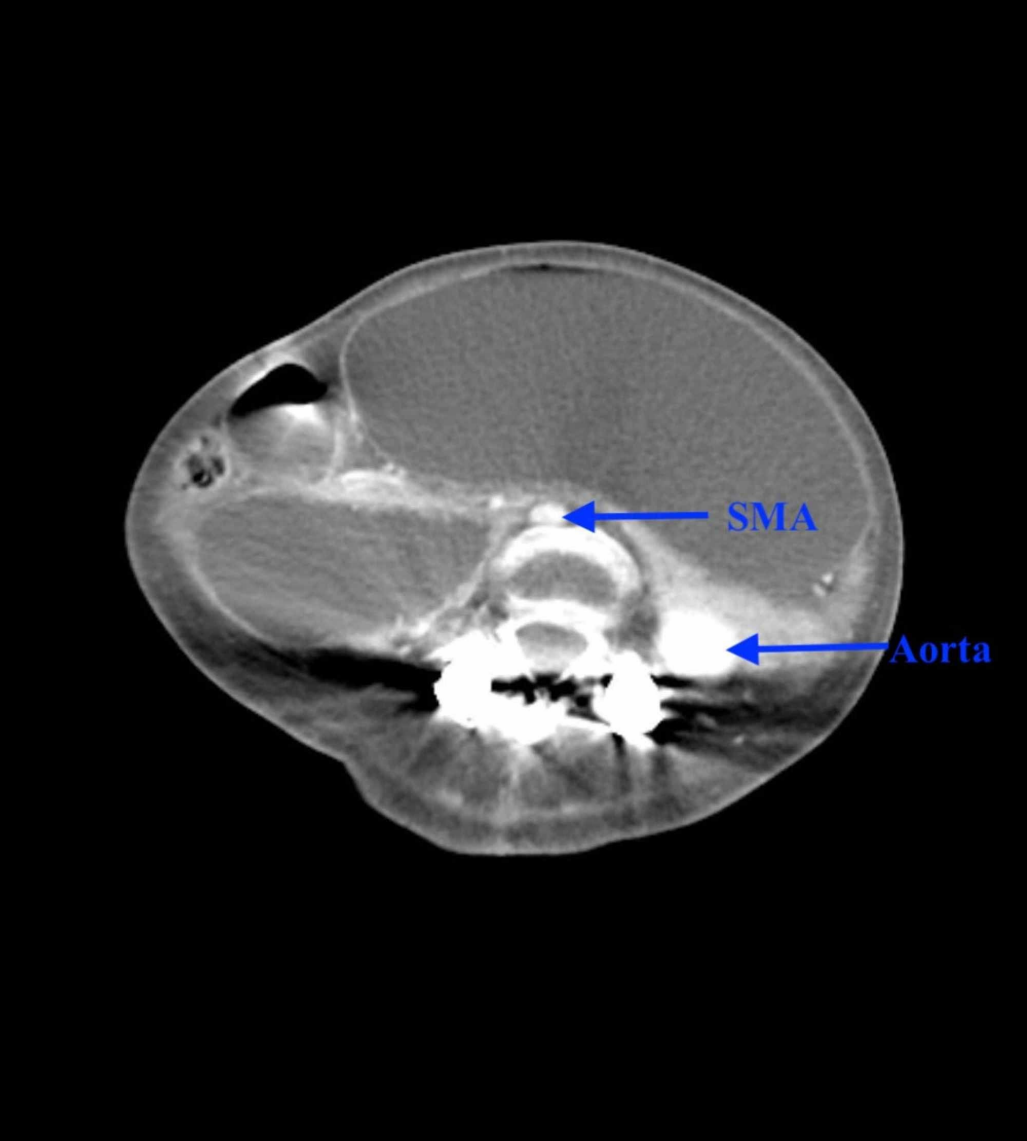
Computed tomography (CT) scan of the abdomen (axial section) with contrast showing distended stomach with distended duodenum to the level of SMA with narrow AMD <1 cm SMA: superior mesenteric artery; AMD: aorto-mesenteric distance.

Computed tomography (CT) scan of the abdomen and pelvis with contrast showed massive gastric distention with the stomach extending down to the low pelvis, and dilatation of the proximal duodenum to the level of the midline/superior mesenteric artery (SMA) (Figures 1-3).

**Figure 4:**
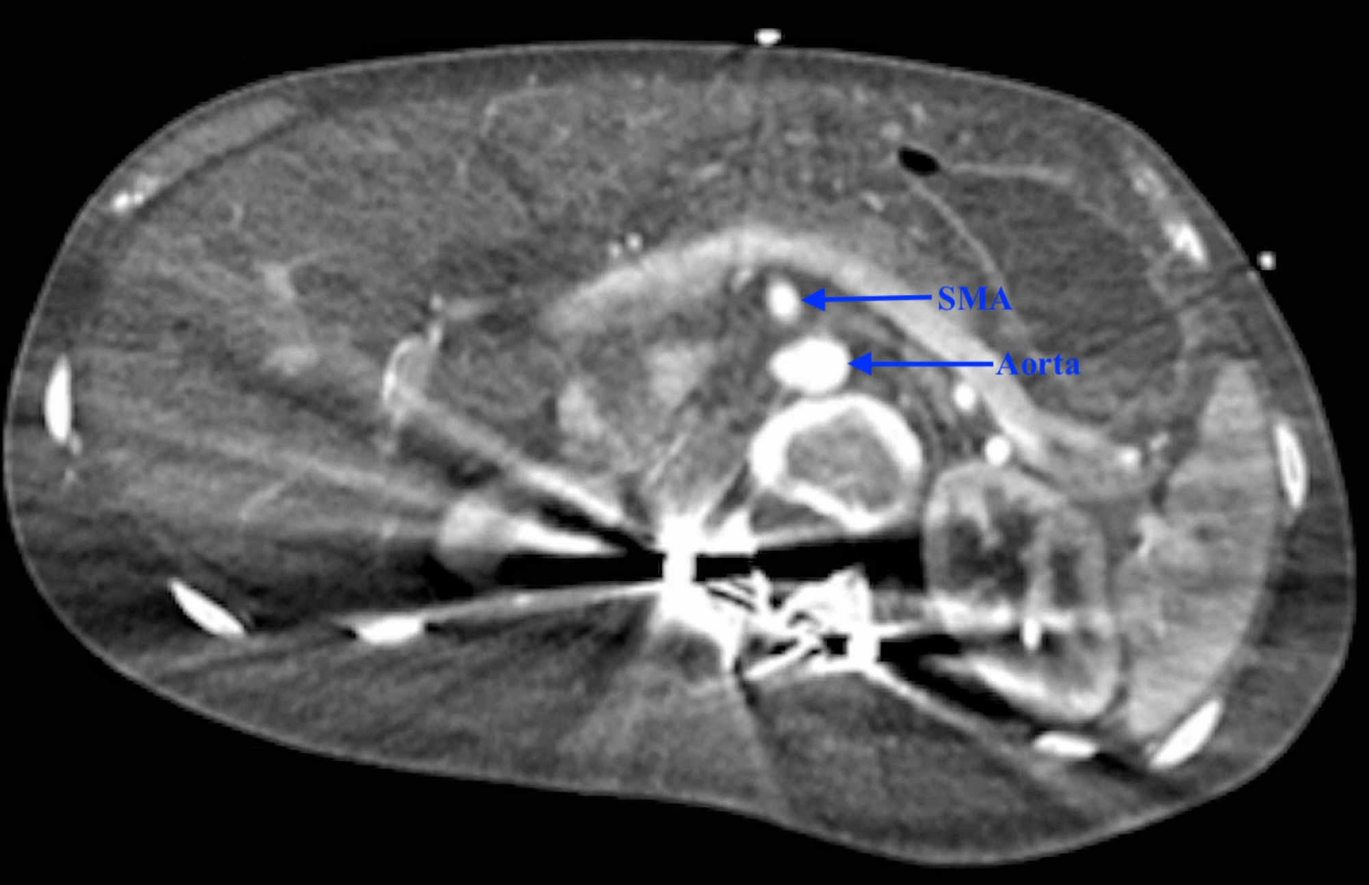
Computed tomography (CT) scan of the abdomen (axial section) with contrast showing resolution of gastric distention with unchanged AMD <1 cm SMA: superior mesenteric artery; AMD: aorto-mesenteric distance.

Since aorto-mesenteric distance (AMD) was less than 1 cm, a working diagnosis of SMA syndrome was made. Upon placement of a nasogastric tube, three liters of gastric bilious contents were evacuated. He was started on lactulose and was given a fleet of enemas with manual disimpaction to help him have bowel movements. Esophagogastroduodenoscopy showed ulcers in the anterior wall and greater curvature of the stomach body, with a normal duodenal bulb and the second/third part of the duodenum. No strictures were visualized during the procedure. CT angiography of the abdomen showed interval resolution of gastric and duodenal distention, ruling out SMA syndrome. The patient’s gastric distention resolved with conservative measures without any need of surgical intervention (Figure 4).

## Discussion

Background

DMD is a progressive, X-linked recessive disorder with an incidence of approximately 30 per 100,000 live-born males. Patients can become symptomatic at 3-5 years of age and become fully wheelchair dependent by 12 years of age [2]. DMD is caused by a mutation in the gene encoding the protein dystrophin. This protein is an important part of the conduction system and affects cell motility, shape, and transport [3-4]. Dystrophin is located on the inner surface of the sarcolemma of muscle fibers and forms protein complexes with glycoproteins, which aid in stabilization. In patients with DMD, this complex lacks dystrophin which leads to a weak sarcolemma. Subsequently, muscle fibers undergo necrosis and are replaced by connective tissue and fat, which can be visualized on muscle biopsy [2].

In patients with DMD, the gastrointestinal tract, which primarily consists of smooth muscle, degenerates and atrophies while the connective tissue infiltrates [5]. Animal studies have shown that dystrophin is present in the smooth muscle layers of the gastrointestinal tract of control animals except for mice lacking full-length dystrophin and maintaining shorter isoforms (mdx mice) [6]. Further studies have shown that mdx mice might have an existing neuropathy in the myenteric neurons leading to gastrointestinal dysmotility of different severity. This might be due to the increased size of synaptic vesicles and the number of recycling vesicles in the myenteric neurons along with the lack of dystrophin protein [7]. Disorders of gastric motility can result from a wide variety of causes and can be divided into: disorders affecting the autonomic nervous system (diabetes, vagotomy), enteric nervous system (visceral neuropathy), smooth muscle (DMD, scleroderma, amyloidosis, mitochondrial cytopathy) and, possibly, abnormalities of the interstitial cells of Cajal (ICC). Our case falls into the category of smooth muscle disorders leading to the gastric hypo-motility. Very few case reports of severe gastric and small bowel dilatation in DMD patients have been reported in the literature. Crowe et al. described the first case of acute gastric dilatation in a 9-year-old boy with DMD in 1961. This was followed by few other case reports by Robin and Falewski et al. (1963), Stark et al. (1988), and Lunshof et al. (2000) [1,8-11].

Gastrointestinal manifestations

Gastrointestinal symptoms that manifest as a result of DMD have garnered more interest in order to more optimally diagnose and treat. Many patients’ life spans do not reach adulthood, leading to a lack of experience in treating adults with DMD. Since smooth muscle is located throughout the gastrointestinal tract, its degeneration affects multiple organs. Dysphagia (36% in 1 series) due to oropharyngeal dysfunction is one of the most predominant symptoms in DMD patients [3]. Gastroparesis leading to gastrointestinal dilatation is another common problem affecting these patients. Gastric emptying times in DMD patients, of various ages, were shown to be significantly longer than their controls [12]. Barohn et al. showed that DMD patients have delayed gastric-emptying times using radionuclide scintigraphy studies [13]. Severe gastroparesis can lead to severe gastric and small bowel dilatation. Impaired colonic transit time, due to lack of ambulation, abdominal muscle weakness, and atrophy of smooth muscle, might lead to high incidences of constipation in these patients [14]. Chronic constipation can further compound the severity of gastric or small bowel dilatation, which can be life threatening. Our patient also presented with this gastroparesis, which was evident by vomiting and massively dilated stomach and small intestine, causing colonic compression. His gastric dilatation was further worsened by his severe chronic constipation. Wheelchair-bound patients can also present with gastro-esophageal reflux disease (GERD) and esophagitis due to deterioration of the lower esophageal sphincter [15].

Treatment of gastrointestinal manifestations

The gastric manifestations in patients with DMD have been poorly understood and thus, under-treated. This holds especially true in the adult population. Because DMD is a progressive disease, adult patients have more extensive muscular deterioration and therefore present with more severe symptoms. Proton pump inhibitors are effective, with few side effects in patients with GERD and esophagitis. Constipation may be treated conservatively with increased fluid and fiber intake or medically using stool softeners, laxatives, and fleet enemas [15]. Studies have proven that pro-kinetics especially aid in treating neuropathic manifestations of DMD [4]. Gastric dilatation may be treated with nasogastric tube decompression, which if done in a timely manner may help restore proper motility in acute cases [4,15]. If healthcare providers become more aware of the existence of gastrointestinal manifestations in patients with DMD, a more direct approach may be used for treatment, which could also be cost effective.

Ruling out other differential diagnoses

The severe gastric and small bowel dilatation in our patient reached the level of the SMA and was found to have a narrowed AMD of less than 1 cm, leading to the inclusion of SMA syndrome. Normal AMD is 1 to 2.8 cm [16]. However, taking into account the patient’s past medical history, conservative measures including nasogastric decompression, stool softeners, laxatives, fleet enemas, and manual disimpaction were performed with drastic resolution of gastric distention on repeat imaging. There was no improvement in the AMD interval on the repeat imaging, which ruled out SMA syndrome as a contributing factor towards the patient’s massive gastric dilatation. The decreased AMD in our patient could have been due to loss of fat, given his BMI was only 14.8 kg/m2.

## Conclusions

This was a unique case of acute gastric and small bowel dilatation, which is a rare manifestation of DMD most likely secondary to gastroparesis. Our patient’s symptoms were due to gastric and small bowel dilatation, which resolved with the conservative management with no need of surgery. Based on our experience, internists should be further educated on gastrointestinal manifestations in adult patients with DMD in order to improve outcomes, as it is a rare entity encountered in adults due to the short lifespan of these patients.
